# The Role of Immune Correlates and Surrogate Markers in the Development of Vaccines and Immunotherapies for Plague

**DOI:** 10.1155/2012/365980

**Published:** 2011-09-29

**Authors:** E. D. Williamson

**Affiliations:** Biomedical Sciences Department, Defence Science & Technology Laboratory, Porton Down, Salisbury Wilts SP4 0JQ, UK

## Abstract

One of the difficulties in developing countermeasures to biothreat agents is the challenge inherent in demonstrating their efficacy in man. Since the first publication of the Animal Rule by the FDA, there has been increased discussion of potential correlates of protection in animal models and their use to establish surrogate markers of efficacy in man. The latter need to be relatively easy to measure in assays that are at least qualified, if not validated, in order to derive a quantitative assessment of the clinical benefit conferred. The demonstration of safety and clinical benefit is essential to achieve regulatory approval for countermeasures for which clinical efficacy cannot be tested directly, as is the case for example, for biodefence vaccines. Plague is an ancient, serious infectious disease which is still endemic in regions of the modern world and is a potential biothreat agent. This paper discusses potential immune correlates of protection for plague, from which it may be possible to derive surrogate markers of efficacy, in order to predict the clinical efficacy of candidate prophylaxes and therapies.

## 1. Plague

The ancient disease of plague is still present in endemic regions of the modern world and results in approximately 3,000 reported cases each year [1]. Plague is a flea-vectored infection caused by the Gram-negative bacterium *Yersinia pestis, *a potential biothreat agent. Originally an enteric pathogen, *Y. pestis* is thought to have evolved from the enteropathogen *Y. pseudotuberculosis *[2] as a flea-vectored, enzootic infection. Fleas feed on infected rodents and then transmit bacteria to a susceptible mammal by flea bite. Man is an accidental host in this cycle, but if bitten can contract bubonic plague, a serious infection if not treated promptly before the individual becomes symptomatic. A secondary pneumonic plague can develop in an individual suffering from bubonic plague, and this is of even greater concern, since *Y. pestis* bacteria are highly transmissible in aerosolised form between unprotected individuals in close contact, with the potential for epidemic spread [3].

## 2. Virulence Factors in *Yersinia pestis*

*Y. pestis* produces a range of antigens and virulence factors, three of which have known protective efficacy as candidate subunit vaccines: F1-antigen [4], V-antigen [5], and Yersinia secretory factor F (YscF) [6]. These three proteins are virulence factors when secreted by *Y. pestis* during infection. F1 antigen is a capsular protein with antiphagocytic properties [7], whilst the V-antigen is a regulatory protein in the type three secretion system (TTS) utilised by the bacterium to gain access to and deliver other cytotoxic and anti-phagocytic Yersinia outer proteins (Yops) to host cells [8]. V-antigen occurs both within the bacterium, where it has some regulatory function in the initiation of the TTS process, and also at the tip of the injectisome [9]. The columnar structure of the injectisome is comprised of YscF [6]. 

Many other factors have been evaluated as potential vaccine candidates, including for example plasminogen activator (Pla), which during infection facilitates the delivery of the blood meal from the infected flea into mammalian host cells, by the degradation of physical barriers such as endothelium and connective tissue [10, 11]. However, Pla was found to be poorly immunogenic and provided no protection against lethal plague in a mouse model [11]. YscF has also been evaluated for efficacy [6], as has ph6 antigen [12] and many of the Yop's [13, 14]. Whilst some of these factors confer partial protection in animal models of plague, full protective efficacy against *Y. pestis* has been achieved only with F1- or V-antigens and was optimum when these antigens were used in combination, or as a genetic fusion [15, 16]. The protective efficacy of the combined recombinant F1 and V (rF1 + rV) subunits against *Y. pestis* has now been reported by a number of laboratories and in a range of laboratory animal models (reviewed in [17]). Immunisation with rF1/V has been shown to protect animal models against flea-vectored plague [18] as well as against experimental exposure to *Y. pestis *[19, 20].

## 3. Vaccination to Protect against Plague

Vaccination and postexposure therapy are both options to protect individuals against exposure to *Y. pestis*. There has been a series of killed whole cell vaccines (KWCVs) for plague, starting with Haffkine's vaccine in the late 1800s [21], through to the currently available KWCV produced by the Central Serum Laboratories (CSL), which comprises a suspension of heat-killed *Y. pestis* (>10^9^/mL). Whilst KWCVs are efficacious against bubonic plague, epidemiological evidence suggests that they have little protective efficacy against pneumonic plague [15, 20, 22, 23]. F1 appears to be the key protective antigen in KWCV formulations, which thus do not protect against F1^−^ *Y. pestis *[4, 5]. Unlike the KWCVs, the rF1-/V-antigen combination has been demonstrated to protect both mice and macaques against pneumonic plague [24–27], representing a significant advance in candidate countermeasures for plague infection. Different presentations of F1/V have been studied including DNA vaccines [28], oral formulations [29], and live vaccine-vectored expression from, for example, salmonella strains [30].

A naturally attenuated live vaccine strain, EV76, has been demonstrated to protect mice and macaque models against pneumonic plague [31, 32]. Recently, a strain of *Y. pestis* KIM, mutated to stimulate TLR4 responses in the vaccinee, has been mooted as a vaccine candidate, protecting 80% of vaccinated mice against pneumonic plague [33]. This differential between live attenuated and killed vaccines in efficacy against pneumonic plague has been attributed to lack of the V-antigen in the KWCV formulations which contain effective quantities of the F1-antigen only [15, 26]; by comparison, live attenuated vaccines contain both F1- and V-antigens [15]. However, live attenuated vaccines such as EV76, have caused morbidity in nonhuman primates (NHP) [34], raising safety concerns over their use in man.

## 4. Postexposure Therapy

The early detection and administration of antibiotic therapy within 18–24 hours following suspected exposure to *Y. pestis* and before the appearance of symptoms, is critical for the successful treatment of plague. The recommended antibiotic regimen comprises a high dose of gentamicin intravenously (5 mg/kg intravenously once a day) or the equivalent dosage of streptomycin, ciprofloxacin, gentamicin, or doxycycline for 10 days [35]. Chloramphenicol may also be used if plague meningitis is suspected [36]. As the patient responds to treatment, it may be possible to change to the oral route of administration of the preferred antibiotic. It is essential that antibiotic treatment is adjusted dependent on the antibiotic susceptibility of the infecting organism in culture, particularly if deliberate use of an antibiotic- resistant strain is suspected. 

In animal models, the administration of monoclonal antibodies (Mab's) with specificity for F1 and V, has been shown to protect mice infected with *Y. pestis*, even when the Mab's were administered at 48 h post-exposure [37]. However, the protective effect of the anti-V Mab 7.3 was abrogated by the coadministration of anti-TNF*α* and anti-IFN*γ* indicating that a cellular proinflammatory response is also contributing to protection [38]. There is scope for combining immuno- and antibiotic therapy after exposure to *Y. pestis*, in order to shorten the duration of antibiotic therapy required.

## 5. Bridging between Nonclinical and Clinical

Since standard Phase III clinical efficacy studies are not feasible to carry out with plague and other serious human diseases, on both ethical and practical grounds (too few naturally occurring cases as well as outbreaks which are spasmodic), it is essential to establish satisfactory animal models of the disease. These, in turn, can be used to assess the efficacy of candidate vaccines and therapies and to identify correlates of protection. Robust animal models of plague infection which authentically represent the human disease syndrome are the objective and models have been established in standard laboratory animal species (mouse, rat, rabbit, and macaque), as well as nonstandard species such as the black-footed ferret (reviewed in [17]). 

The rF1/V combination is potently immunogenic in the mouse, guinea pig, macaque, and human [15, 39–41] and has been shown to be efficacious in nonclinical models against either injected [15], aerosolised [16, 20], flea-vectored [18], or ingested [42] exposure to *Y. pestis*. From these nonclinical studies, there is a need to identify the immune correlates of protection to facilitate the progression of candidate countermeasures through the clinical phase. 

**Figure 1:**
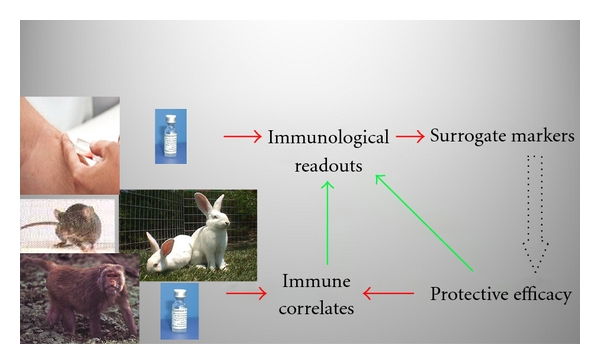
Integrating immunological readouts from nonclinical and clinical studies to identify surrogate markers of efficacy.

Bridging the gap between the nonclinical and clinical phases of the development process for a countermeasure is arguably the most risky element of the entire R&D cycle and has previously been termed “the valley of death” [43]. Many candidate prophylaxes and therapies have foundered at this interface, possibly because of the difficulty in comparing nonclinical and clinical datasets in terms of protective efficacy. This highlights the need to understand the immunological mechanisms required to achieve protective efficacy against such agents and to derive immune correlates of protection in animal models. Identification of the latter, based on immunological readouts which have been found to correlate statistically with protective efficacy in appropriate animal models, should lead to the derivation of surrogate markers of efficacy (Figure 1). Surrogate markers need to be measurable and quantitative endpoints for clinical trial volunteers which predict efficacy. If several surrogate markers are used, collectively these may be used to predict the degree of efficacy that can be achieved. Thus the nomination of surrogate markers of efficacy effectively bridges the gap between the nonclinical and clinical phases of R&D.

Depending on how closely the animal model mimics the human infection, more than one animal model of the infection may be required to provide immune correlates, concepts embodied in the Animal Rule by the Food and Drug Agency in the USA [44]. In summary, the Animal Rule requires the following:

1. there is a well-understood pathophysiological mechanism operating and of its prevention or substantial reduction by the product;
2. the effect is demonstrated in one or more animal species expected to react with a response predictive for humans, unless the effect is demonstrated in a single animal species that represents a sufficiently well-characterized animal model for predicting the response in humans;
3. the animal study endpoint is clearly related to the desired benefit in humans, generally the enhancement of survival or prevention of major morbidity;
4. the data or information on the kinetics and pharmacodynamics of the product in animals and humans, allows selection of an effective dose in humans.

Having identified immune correlates of protection, there are various mathematical approaches to extrapolate these nonclinical data to man in order to predict degrees of protection [45].

## 6. Immune Correlates of Protection in Plague

Immunisation of mice with either [4, 5] or both F1 and V proteins [15, 16] was protective against plague and a titre of specific antibody correlated with protection. Whilst the development of an IgG titre to these proteins correlates with protection as observed in mice [46], guinea pig [39], nonhuman primate (NHP) [40] and inferred from passive transfer studies with clinical trial serum [41], neutralizing antibody alone does not describe the entire mechanism of protection against this virulent pathogen [17]. Researchers from several groups have reported a strong CMI response to be operating [38, 47, 48] and in response to an alhydrogel-adsorbed formulation of the rF1 + rV vaccine, this generally has been observed to be a CD4+ Th2-biased CMI response [46]. However, alternative formulations of the rF1 + rV vaccine in which different adjuvants have been substituted for alhydrogel have also been demonstrated to induce protective immunity in a CD4+ Th1-biased setting [49, 50]. Additionally, strains of mice with targeted gene deletions affecting antibody production by B cells (*μ*MT B cell knockouts or SCID/beige) or the nature of the Th cell response including Stat 4/Stat 6 knockouts and IL4/IL10 knockouts have been studied [17, 49–51]; rF1 + rV-immunised Stat-4-deficient mice, which have low levels of IFN*γ* production, were found to be poorly protected from *Y. pestis* challenge, despite producing similar antibody titres to rF1 + rV as the intact controls [49]. Moreover, the rF1 + rV vaccine was able to induce protective immunity in IL4 knockout mice despite a Th1-biased environment operating in these animals [50]. Indeed, Stat-4-mediated immune mechanisms leading to a Th1 response were found to be essential for protection, whereas Stat 6/Th2-mediated responses were not [49]. Thus for the rF1 + rV vaccine, the induction of specific antibody neutralising the F1 and V antigens is a significant immune correlate of protection; however the supporting CMI response is not necessarily Th2-polarised and indeed the operation of Th1 mechanisms during infection appears to be essential for full protection and recovery [17].

**Figure 2:**
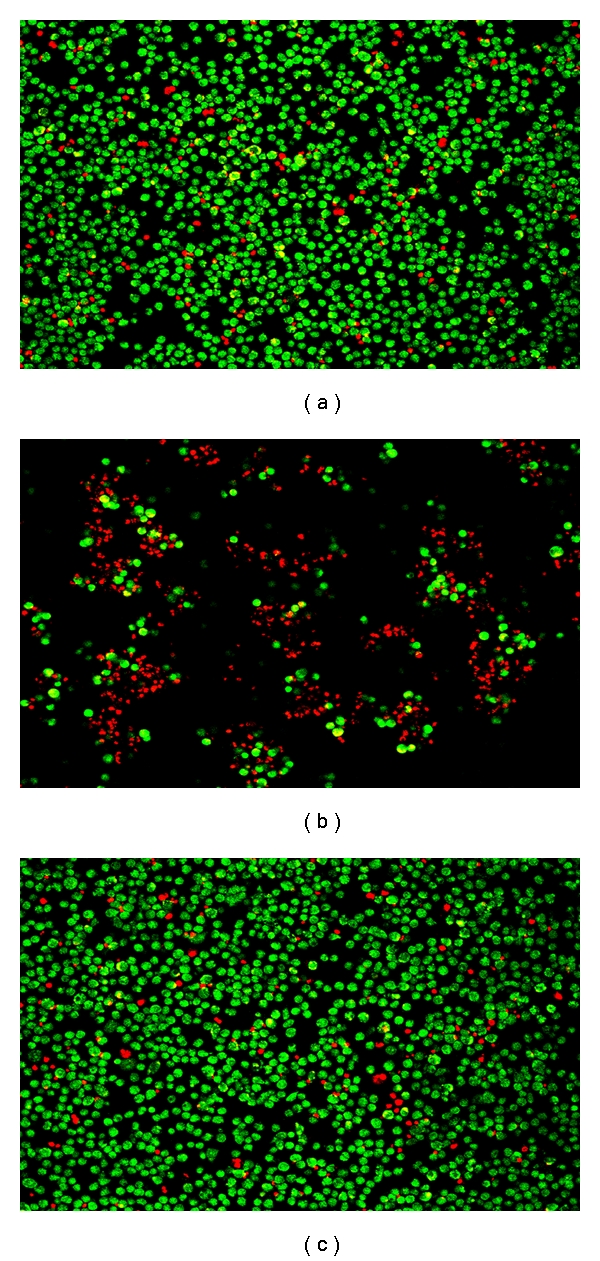
The cytoxicity of *Y. pestis* V antigen expressed from *Y. pseudotuberculosis* for J774 cells was inhibited by pretreatment with anti-V Mab 7.3. Cells were stained with ethidium bromide/acridine orange to identify live cells (green) and dead cells (red). (a) Unifected J774 cells, (b) J774 cells infected with *Y. pseudotuberculosis* expressing V antigen were killed, shown by the preponderance of dead cells. (c) J774 cells pretreated with Mab 7.3 prior to exposure to *Y. pseudotuberculosis* expressing V antigen were protected, with no significant difference in appearance, compared with uninfected cells.

Whilst the measurement of total Ig indicates that an immune response has been induced by a candidate vaccine, this alone cannot indicate that protective immunity has been achieved. The assay of the functionality of the induced antibody may be more instructive. If protection can be demonstrated in the selected animal models and related to the presence of a neutralising antibody response, then the identification of the same neutralising antibody within serum samples from human clinical trial volunteers indicates an immune correlate of protection and potential surrogate marker of efficacy. Thus immune macaque [40] and human Phase 1 trial volunteers [41] sera have been demonstrated to compete with the plague-protective monoclonal antibody (Mab 7.3) for binding to the V-antigen on solid phase *in vitro;* these sera (results not shown) as well as Mab 7.3 protected J774 cells *in vitro* from the cytotoxic effect of V-antigen secreted by *Y. Pseudotuberculosis* (Figure 2); passively protected naive mice from *in vivo* challenge with *Y. pestis*. The passive transfer of protective immunity in human serum into mice also correlated significantly with the total IgG titre in the human donors to rF1 + rV at days 21 (*P* < 0.001) and 28 (*P* < 0.03) [41].

 Subsequently however, competitive ELISA has not been shown to be consistent between laboratories as a correlate of protection assay [52], likely due to the existence of more than one protective B-cell epitope on the V antigen [53]. Thus a pragmatic approach towards assays showing a correlation between immunological readouts in relevant animal models and man needs to be taken to thoroughly test such assays for consistency and utility. 

## 7. Potential Surrogate Markers of Efficacy for Countermeasures to Plague

Based on these data on immune correlates and on the immunoanalysis data published to date on samples from clinical trial volunteers immunised with the rF1/V subunit vaccine [41], it is possible to identify several serological surrogate markers of efficacy. These may include the inhibitory-activity of human immune serum on the cytotoxicity of V-antigen secreted from *Y. pseudotuberculosis.* Qualitative data from this assay have been published [40], however, the assay has subsequently been improved and made quantitative [54]. It has been demonstrated that decreased caspase-3 activity in macrophages exposed to immune NHP serum correlated with increased survival of those NHP to *Y. pestis* infection.

Passive transfer of human serum from volunteers enrolled in a Phase I clinical trial has been demonstrated to protect naive mice against plague infection, in a dose-related manner [41]. The passive transfer of protective immunity into mice also correlated significantly with total IgG titer to rF1 plus rV at days 21 (*r* ^2^ = 98.6%; *P* < 0.001) and 28 (*r* ^2^ = 76.8%; *P* < 0.03). 

Assays for cellular surrogate markers of efficacy have traditionally been more challenging, particularly in a clinical setting, since they have required fresh whole blood samples and relatively prompt analysis. However recent advances in flow cytometry have simplified this, allowing the assay of T-cell responses and the quantitative analysis of lymphocyte subsets in whole blood. Nevertheless, sample size is important: attempts to analyse changes in cell surface markers on peripheral blood mononuclear cells (PBMC) by flow cytometry during the course of a small Phase 1 clinical trial for rF1V did not reveal any significant trends, due to the large variation in response between individuals [41]. The demonstration of a cellular recall response to rF1/V has been reported in *ex vivo* splenocytes from immunised mice [55]. A more practical alternative may be to use an ELIspot assay, where for example, IFN*γ* secretion from splenocytes restimulated *in vitro* with vaccine antigens is detected [48]. More specifically, CD4+ T-cell epitopes for F1 and V have been identified in mice [56, 57] and an H-2^d^-restricted murine T-cell epitope in F1 has been shown to be essential for protection in Balb/c mice [58]. Similarly, HLA-restricted T-cell epitopes have been mapped in F1 [59] and are being sought in V-antigen using HLA transgenic mice. These data may in the future provide functional targets for human T-cell memory responses, recognition of which by immune PBMC could provide a cellular surrogate marker of efficacy.

## 8. Conclusions

Much work is ongoing to identify statistically valid immune correlates of protection for plague, particularly since a clinical demonstration of efficacy is not possible. This has required the development of nonclinical models which authentically represent the human infection. As far as possible, the immune correlate should be demonstrated in more than one nonclinical model. Whilst the immune correlate(s) may not describe all the immune mechanisms operating in protection against a pathogen, they should be reproducibly consistent between the selected nonclinical models and the clinic and should be quantitative, to assess the likely benefit to be conferred on the vaccinee. With an increasing understanding of the molecular basis of pathogenicity and of the innate and adaptive immune response mechanisms required to counter *Y. pestis*, immune correlates of protection are being identified and reported and this in turn will expedite the development of next-generation vaccines and immunotherapies.
